# Losing DNA methylation at repetitive elements and breaking bad

**DOI:** 10.1186/s13072-021-00400-z

**Published:** 2021-06-03

**Authors:** Xena Giada Pappalardo, Viviana Barra

**Affiliations:** 1 Department of Biomedical and Biotechnological Sciences (BIOMETEC), University of Catania, 95125 Catania, Italy; 2 National Council of Research, Institute for Biomedical Research and Innovation (IRIB), Unit of Catania, 95125 Catania, Italy; 3 Department of Biological, Chemical and Pharmaceutical Sciences and Technologies (STEBICEF), University of Palermo, 90128 Palermo, Italy

**Keywords:** DNA hypomethylation, Repetitive DNA, Satellites, LINE-1, Cancer, ICF syndrome, Autism spectrum disorder, Alzheimer’s disease, Neuropsychiatric disorders, Hereditary diseases

## Abstract

**Background:**

DNA methylation is an epigenetic chromatin mark that allows heterochromatin formation and gene silencing. It has a fundamental role in preserving genome stability (including chromosome stability) by controlling both gene expression and chromatin structure. Therefore, the onset of an incorrect pattern of DNA methylation is potentially dangerous for the cells. This is particularly important with respect to repetitive elements, which constitute the third of the human genome.

**Main body:**

Repetitive sequences are involved in several cell processes, however, due to their intrinsic nature, they can be a source of genome instability. Thus, most repetitive elements are usually methylated to maintain a heterochromatic, repressed state. Notably, there is increasing evidence showing that repetitive elements (satellites, long interspersed nuclear elements (LINEs), Alus) are frequently hypomethylated in various of human pathologies, from cancer to psychiatric disorders. Repetitive sequences’ hypomethylation correlates with chromatin relaxation and unscheduled transcription. If these alterations are directly involved in human diseases aetiology and how, is still under investigation.

**Conclusions:**

Hypomethylation of different families of repetitive sequences is recurrent in many different human diseases, suggesting that the methylation status of these elements can be involved in preservation of human health. This provides a promising point of view towards the research of therapeutic strategies focused on specifically tuning DNA methylation of DNA repeats.

## Background

DNA methylation (DNAm) is the first epigenetic modification discovered in humans, which is implicated in a variety of cellular processes in mammals including cell differentiation, genomic imprinting, and X-inactivation [1]. DNAm refers to the covalent addition of a methyl group to the C-5 position of the cytidine at the 5’ of the CpG dinucleotide, mediated by dedicated enzymes called DNA methyltransferases (DNMTs) [2]. 5-Methyl-cytosine (5mC) is very common in the eukaryotic genome that it is considered a fifth base [3]. Moreover, its pattern is established during development by de novo DNMTs (DNMT3a and 3b in humans) and preserved by the maintenance methyltransferase DNMT1 [2]. During cell division, the global CpG methylation pattern is faithfully maintained in daughter cells through the action of maintenance DNMT1 that interacts with the proliferating-cell nuclear antigen (PCNA), a cofactor of DNA polymerase delta, and the ubiquitin-like containing plant homeodomain and RING Finger domains 1 (UHRF1), a component of the DNA replication fork [4, 5]. UHRF1 preferentially recognizes the hemi-methylated CpGs, generated during DNA replication, (or the H3K9me2/3) where it targets DNMT1 activity [5, 6]. Notably, DNAm is essential for development since the loss of any of the three DNMTs has been shown to be lethal for mice [7, 8]. Moreover, during human embryonic development, an epigenetic programming of DNAm models a cell specific signature of gene expression [9–11]. Also, the exposure to various nutritional and environmental signals early in life may influence the methylation dynamics of all methylated segments of the genome, potentially affecting the developmental trajectories of both healthy and disease states [12].

The distribution of DNAm along different genomic segments influences the functional assembly of chromatin, involving other epigenetic modifications such as histone modifications and nucleosome positioning to modulate transcriptionally active or inactive state in specific biological contexts. However, the regulatory interactions influencing the variation of DNAm levels and the chromatin folding need to be fully elucidated [13, 14].

Methylated cytosines induce tighter wrapping of DNA around the nucleosome histone core [15] and are binding sites of Methyl-CpG binding proteins which recruit repressive epigenetic factors [16]. In this way, DNAm contributes to chromatin condensation and gene silencing.

A peculiar aspect of epigenetic modifications, including DNAm, is their dynamic and reversible nature. However, the direct erasure of 5mC would be an energy demanding process and it has not been observed in vivo. Instead, multistep processes have been described to induce active DNA demethylation. These involve firstly the modification of the 5mC through oxidation or deamination performed by ten-eleven translocation (TET) and activation induced deaminase (AID) proteins, respectively. Then, the base excision repair (BER) intervenes to replace the modified nucleotide (for review on active DNA demethylation see [17]).

By changing the degree of compaction of the chromatin, DNA methylation is known for its role in transcriptional repression. As a matter of fact, the so-called CpG islands, regions rich in CpG dinucleotides that are protected from methylation, are embedded in most gene promoters. The activation of such promoters is therefore highly regulated, and alterations of this regulation by unscheduled methylation correlate with abnormal repression of these genes, a condition frequently observed in cancer cells [18].

Despite the importance of transcriptional regulation, CpG islands of gene promoters are merely a small part of the genome whose methylation status can be changed. On the contrary, the majority of CpG dinucleotides is found in repetitive DNA (repetitive elements, REs) [19], the most abundant type of sequences in the genome. The first sequencing of the human genome surprisingly unveiled that almost half of it is made up of repetitive sequences [20]. One decade later, De Koning et colleagues, by using a newer computational strategy, inferred that in fact more than two third of the genome consists of REs [21]. Far from the traditional concept of coding sequences, REs have been considered for a long time as “junk” DNA [22]. However, taking into account the amount of these sequences in our genome, a more reasonable speculation has risen, that REs are elements of the genome whose functions we still have to fully disclose.

REs are distinguished into two classes: tandem and interspersed repeats. Tandem repeats are composed by one or more nucleotides repeated in a block or an array in a head-to-tail orientation and are usually non-coding sequences. According to the size of the repeated unit and the total length, they can be further classified in satellites (sat1, sat2, sat3, centromeric alpha-satellites, telomeres), minisatellites (variable number of tandem repeats (VNTRs)) and microsatellites (simple sequence repeats, SSRs). A common characteristic of tandem repeats is their instability, intended as variability in the number of repeats, which, in turn might affect genome stability [23]. Tandem repeats contraction/expansion has been demonstrated to be induced by replication slippage and recombination due to the high repetitiveness of these sequences [24, 25].

Interspersed repeats are repeated sequences wherein the single copies are scattered in the whole genome with the capability to move from a genomic site to another (transposable elements (TEs), such as LINE-1 and Alu repeats) [26, 27]. However, most of TEs are inactive and lose their mobility as a result of several evolutionary defence mechanisms, among which the main ones concern the maintenance of chromatin condensation to limit DNA accessibility and the accumulation of mutations to eliminate ORFs or inactivate translated proteins (for review see [28, 29]). It is noteworthy that both active and inactive TEs can give rise to genomic rearrangements by transposition or by recombination at distant homologous genomic positions [30].

**Fig. 1:**
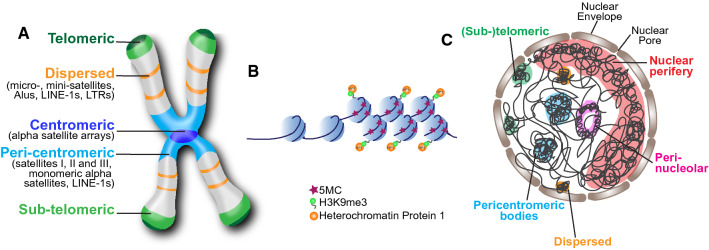
Characteristics and localization of repetitive DNA. **A** Distribution of repetitive elements along the chromosome. **B** Schematics showing the epigenetic characteristics of heterochromatin with methylated cytosines and nucleosomes containing histone H2 di- or tri-methylated on lysine 9 (H3K9me3). Heterochromatic protein 1 (HP1) binds H3K9me3. **C** Schematic representation of the nucleus showing the organization and localization of heterochromatic REs which are mainly distributed in the nuclear periphery, in the perinucleolar space or as heterochromatic bodies like the case of pericentromeres

REs are distributed along the entire chromosome and compose essential parts of it, such as the centromere and the telomere which have both a functional and a structural role (Fig. 1A). The contribution of REs to chromosome 3D structure has been recently shown by using a computational approach that analysed previously published data of chromosome conformation capture experiments (Hi-C). The results of this analysis highlighted the association between the folding of the metazoan genome, including human genome, and the colocalizing of the different types of REs [31]. This finding strongly supports the modern theory that REs direct chromosome packaging by pairing them with their homologous regions and forming compact repeat assemblies which drive the high-order chromosome organization [32].

Rather than parasite or junk DNA, as previously thought, REs play beneficial roles in cells. In fact, centromeres/pericentromeres and telomeres, whose structures and functions are essential for genome stability, are made up of REs. REs are also required for genome replication, which is seen at centromeres (discussed previously in [33]), they are also responsive to environmental signals (reviewed in [34]) and thus most probably serve as a means for adaptive evolution. In addition, REs contribute to the regulation of gene transcription [35, 36], VDJ recombination [37] and nuclear architecture [35].

However, no benefit, unfortunately comes without risks. Indeed, repetitive sequences have intrinsic characteristics that make these regions unstable. Being non-unique, REs (both tandem and interspersed repeats) can easily recombine between them all-over the genome, thus leading to chromosomal rearrangements such as deletions and insertions, translocations and inversions whose effect can be dangerous in living cells (reviewed in [38]). In addition, if the recombination happens during meiosis of germ-line cells it will most likely contribute to diseases. Also, due to their intrinsic mobile nature, TEs can insert genome-wide—with a preference for AT-rich sequences in the case of LINE1 [39]—causing genomic instability either by insertions or genomic rearrangements. Also, the replication of REs can be a source of mutations. Accurate replication of repetitive DNA can be challenging for the replication machinery owing to the many repeats of similar sequences that make it difficult for the replication to progress without errors. Repeats also tend to form secondary structures which constitute an impediment for the proper progression of the replication fork. These problems can induce replication fork stalling that eventually leads to DNA polymerase slippage, incomplete duplication, repeat expansion, breaks and chromosome fragility [40]. Importantly, transcription of REs can also affect the replication of repetitive DNA, by the collision between replication and transcription forks and the formation of DNA:RNA hybrids (R-loops) formed upon annealing of nascent transcripts to one strand of duplex DNA [41–43]. Moreover, it has been shown that overexpression of centromeric alpha-satellites induces mitotic errors and segregation defects in human and murine cell lines [44], though a certain degree of transcription of these REs is necessary for the correct establishment and function of the centromere (reviewed in [33]). Furthermore, it should not be forgotten that transcription activates the retrotransposons, fostering genome instability [29].

In summary, REs could be a source of a multitude of problems that can jeopardize genome stability and result in diseases. Yet, REs’ preservation suggests that the genotoxic effects of REs are balanced against the benefits that they produce (i.e. the centromere, which is made up by REs, is essential for chromosome segregation). To sustain this antagonistic co-evolution, cells have “domesticated” TEs to carry out cell tasks [45] and have evolved epigenetic defence mechanisms to suppress the deleterious effects of REs. Among these mechanisms there are the heterochromatic silenced state (Fig. 1B) (reviewed in [46]), post-transcriptional inhibition by RNA-interference, and the piRNA pathway [47] (for review see [28]). Heterochromatin, thus, is essential to preserve RE functions and maintain their protection [28, 46, 48, 49]. Moreover, in humans, heterochromatin domains are located in specific compartments of the nucleus at the nuclear periphery, perinucleolar bodies and pericentromeric bodies [50], thus reducing the possibility of recombination with other genomic regions (Fig. 1C). Heterochromatin is characterized by specific epigenetic modifications such as H3K9 trimethylation (H3K9 me3) and, as discussed above, DNA methylation (Fig. 1B). Hence, DNA methylation loss or hypomethylation can affect the heterochromatic state of REs and challenge their function and stability.

In fact, alteration of the epigenetic pattern of repetitive sequences is a characteristic of many complex diseases, though it is difficult to understand if it is the cause or the consequence of the disease. However, there are hereditary diseases whose unique known genetic mutation is on DNMT genes with consequent methylation loss at several repetitive elements. This suggests that hypomethylation of REs could have, by itself, the potential to cause a disease in somatic cells. In order to highlight the importance of DNAm at REs, here we will explore human diseases, hereditary and not, in which repetitive elements are hypomethylated and we will analyse the causes and the consequences of this alteration.

## RE hypomethylation in hereditary diseases

### Immunodeficiency with centromeric instability and facial anomalies syndrome

The Immunodeficiency with centromeric instability and facial anomalies syndrome (ICF) (OMIM no. 242860) [51] is an extremely rare autosomal recessive disease (< 70 patients known worldwide) characterized by severe defects in humoral immunity leading to recurrent infections which often result in death at a young age. Patients also exhibit mild facial dysmorphisms, variable intellectual disability and neurological defects. It is cytogenetically characterized by the decondensation of juxtacentromeric regions on chromosomes 1, 9, 16 and rarely 2, which leads to an increased frequency of multibranched chromosomes containing 3 or more arms joined near the centromere. The first observations of the multibranched chromosomes date back to the late 70 s [52, 53]. These peculiar chromosomal rearrangements were reminiscent of the alterations induced by the massive DNA demethylation due to 5 azacytidine (Aza) treatment which drove the researcher to study the methylation pattern of ICF patients. It was, thus, discovered that satellite 2 and 3 were demethylated, and not alpha-satellites, and the inactive chromosome X (Xi) of women [54–58]. Only 2 decades later the discovery of the ICF syndrome, mutations in the *DNMT3B* gene were identified as the cause of the disease, making it the first genetic disease induced by DNA methyltransferase dysfunctions [59]. Actually, *DNMT3B* mutations account for only 55% of ICF patients (ICF subtype 1, ICF1), while the remaining patients carry mutations in *ZBTB24* (*Zinc Finger And BTB Domain Containing 24*) (ICF2) [60], *CDCA7* (*Cell division cycle-associated protein 7*) (ICF3) or *HELLS* (*Helicase, Lymphoid Specific*) (ICF4) genes [61], none of which, when transcribed, give a DNA methyltransferase, but probably a related protein. All the ICF subtypes share the characteristic hypomethylation of pericentromeres. However, they have been clustered into two groups, through the analysis of the Infinium methylation array of 15 patients, based on the different methylation status of other genomic loci [62]. ICF subtypes 2–4, indeed, show hypomethylation of other repeats including alpha-satellites, a feature that was previously observed in few ICF patients [63]. This is not unexpected for ICF4 considering that HELLS, more than 15 years ago, was pointed as an “epigenetic guardian of the repetitive elements” for its role in the protection of repeats from transcriptional activation [64]. Hypomethylation of DNA repeats—which are usually in a heterochromatic state—results in their decondensation on the chromosome. Intuitively, mitosis could be the cell phase where this epigenetic alteration causes problems. In mitosis, chromosomes must be highly condensed to allow the faithful segregation. It has been shown that heterochromatic protein 1 (HP1) protein, which usually binds heterochromatin from the G1 to the G2 phase of the cell cycle, persists through G2 and mitosis in ICF cells as one or two big foci colocalizing with the pericentromeric satellites of chromosomes 1 and 16. This suggested that HP1 serves as an inducer (or controller?) of chromatin condensation and thus remains at hypomethylated pericentromeres in ICF cells that are refractory to condensation even in mitosis [65]. Therefore, DNA hypomethylation (DNAh) certainly induces instability in the affected REs and could underlie the mitotic errors frequently observed in cultured ICF fibroblasts, such as interphase chromatin bridges, lagging chromosomes, micronuclei, anaphase bridges and telomere associations [66].

Telomere fusions are usually possible because of the telomere uncapping and shortening, both characteristics that have been observed in ICF1 cells. In addition, these cells showed hypomethylation of subtelomeric repeats, although not equal for all the chromosomes [67], it correlated with subtended long noncoding RNAs (lncRNAs) transcription, shorter telomeres and premature cell senescence [68–70].

Apart from peri-/centromere and subtelomere repeats, there are also two macrosatellite repeats that have been found hypomethylated in ICF patients, namely D4Z4 and NBL2. D4Z4 and NBL2 have been linked to Facio-Scapulo-Humeral muscular Dystrophy (FSHD) and some types of cancer, respectively (see next paragraphs) [71].

Hypomethylation can allow unscheduled transcription and this happens in ICF cells where massive levels of expression of the hypomethylated repeats have been observed [70, 72–74]. The effects of the transcription of all these loci have not been fully elucidated yet. However, we can speculate that it, most probably, undermines genome stability through defects in centromere and telomere establishment and function, contributing to the short-term survival of the ICF patients. It is noteworthy to mention that, in the zebrafish model of ICF2, the production or just the inoculation of transcripts derived from the pericentromeres triggers an immune response stimulated by interferon [74]. This could correlate with the overexpression of miRNAs with immune function observed in ICF cells [75]. These data suggest that RE hypomethylation is recognized as a threat just like viruses.

Replication timing of hypomethylated pericentromeric satellites is also affected in ICF cells. Indeed, it has been observed, by 5-bromo-2'-deoxyuridine (BrdU) staining and cell fractionation in each cell cycle phase, that Satellite 2 (sat2) is replicated earlier in ICF than in healthy cells. While healthy cells replicate sat2 from late S-phase up to G2/mitosis, ICF cells replicate this region earlier, in mid-S-phase [57]. Moreover, fork progression speed is slightly increased and replication timing shortened [76]. These alterations of DNA replication timing, due to hypomethylation of pericentromeric repeats, can contribute to the chromosomal defects and mitotic errors typical of ICF syndrome.

The massive demethylation found in ICF cells also results in a change of the 3D structure, and positioning of REs. 3D-FISH (fluorescence in situ hybridization) experiments showed that the pericentromeric heterochromatin of chromosome 1 and 9 is markedly closer to the centre of the interphase nucleus in ICF cells than in normal cells [77]. Additionally, Xi and Y chromosomes in ICF cells show a different higher order of chromatin organization, having the genes outside the chromatin domain and not inside or at the edge as in normal cells [78]. We do not exclude that even all the chromosomes have a totally different organization with respect to a healthy condition, and that this alteration can contribute to the genomic instability observed in ICF syndrome.

### Facioscapulohumeral muscular dystrophy syndrome

The facioscapulohumeral muscular dystrophy (FSHD) (OMIM 158,900) is a rare autosomal dominant myopathy (1:20,000 affected individuals worldwide), highly variable, and characterized by progressive, often asymmetric, muscle weakness of the facial, shoulder and upper arms initially, and later also of the abdomen, lower limbs and feet (reviewed in [79]). Intriguingly, no structural mutation in a disease gene has been found. Instead, the patients mainly show DNA rearrangements in the subtelomere of chromosome 4q (specifically 4q35) where variable contractions (reduction of copy number) of the D4Z4 macrosatellite repeat array are present [80]. D4Z4 array is highly polymorphic in the healthy population ranging between 11 to 200 units (3.3 Kb each). In FSHD patients, the number of the units falls down to less than 10 on one allele. D4Z4 is present with high similarity within chromosome 10q, however here any contraction does not cause a disease (reviewed in [81]). Surprisingly, the induced monosomy of 4q did not result in the disease [82]. In addition, phenotypic FSHD patients (FSHD2) without contractions of D4Z4 array have been identified [83]. This evidence suggested that the origin of the disease could lie in the position and the epigenetic features of the locus.

To have further insight on this possibility, in 2003 van Overveld et al. analysed the methylation status of chromosome 4q35 in many tissues of healthy and FSHD individuals at different ages. They observed that in fact FSHD patients including FSHD2 show hypomethylation of the aforementioned DNA region [84] regardless of the age and tissue. Importantly, FSHD2 patients frequently exhibit mutations in the *structural maintenance of chromosomes flexible hinge domain-containing 1* (*SMCHD1*) gene and sometimes mutations in *DNMT3B* (mutations not correlated with ICF syndrome; see the related paragraph above) which are most probably responsible for the methylation loss at D4Z4 repeats [85, 86]. In addition, the analysis of a cohort of patients with variable symptoms highlighted that the degree of methylation loss of chromosome 4q35 correlates with the severity of the disease [87]. These discoveries, clearly, suggested a wider scenario to understand the molecular basis of the pathogenesis of FSHD. Given the similarity with another known syndrome, ICF, the pericentromeric satellites were also analysed in FSHD patients, but no differences in the methylation status has been observed with respect to healthy individuals [88]. Although an accurate genome-wide analysis of DNA methylation has not been performed yet in FSHD patients, no major alterations of heterochromatic regions along the chromosomes has ever been observed except when FSHD is coupled with *DNMT3B* mutations (see above) [86, 88].

Due to the usual strong correlation between DNA methylation and transcription, several studies have been focused on the research of transcriptional alteration from D4Z4 and its proximal region. As expected, the loss of methylation on D4Z4 repeats resulted in the expression of *DUX4* (double homeobox 4 gene), the ORF within each repeated unit, of several RNAs with different functions and of genes in the proximal regions, namely *FRG1*, *FRG2*, *TUBB4q* and *ANT1* [89–93]. Although each of these transcripts could be responsible for muscle degeneration, there is consensus among the researchers in the field that the expression of *DUX4* is at the origin of the disease. Indeed, *DUX4* itself functions as transcriptional activator [89] and FSHD arises only when this polypeptide is produced. Remarkably, up to now, nine different haplotypes of chromosome 4q have been described on the basis of the sequence variation among the population, interestingly only one, the 4qA161, has been correlated with FSHD disorder [94, 95] and with the production of a stable *DUX4* transcript [96].

The regulation of *DUX4* expression is finely regulated by DNA methylation. It has been demonstrated that the hypomethylation of D4Z4 repeats allows the transcription of lncRNA from DBE (D4Z4 binding element) in FSHD patients. This lncRNA localizes to *DUX4* and can recruit proteins of the Trithorax group which, in turn, counteract the repression on *DUX4* [97], thus allowing transcription.

The genome-wide replication profiles of FSHD myogenic precursors have also been analysed, but no significant difference was highlighted in comparison to normal cells [98]. However, up to now, the replication fork speed specifically at D4Z4 repeats has not been studied. Thus, we cannot exclude that hypomethylation of this region affects DNA replication in FSHD cells and that this alteration can contribute to the pathogenesis of the disorder.

### Hereditary sensory neuropathy-sensorineural hearing loss-dementia syndrome

The hereditary sensory and autonomic neuropathy type 1E, (HSAN1E) (OMIM 614,116) is a rare autosomal dominant neurological disorder (< 1:1,000,000 affected individuals worldwide) characterized by progressive degeneration of the central and peripheral nervous systems. This disorder clinically manifests as loss of hearing and early onset of dementia. The neurologic defects appear at 20–35 years of age and progressive cognitive impairment arises by the fourth decade, with death occurring in the fifth/sixth decade [99]. By linkage analysis carried out on the samples of some HSAN1E patients, a region on the chromosome 19p13.2, corresponding to *DNMT1* gene was identified [99]. HSAN1E patients have non-synonymous mutations in the Targeting Sequence (TS) of the N-terminal and the regulatory region of DNMT1. TS is essential for DNMT1 activity, and is responsible for the binding of DNMT1 to chromatin during late S and its persistence in G2 and mitosis. TS also allows the binding of UHRF1 (ubiquitin like with PHD and ring finger domains 1) which is necessary to target DNMT1 to its substrates [100]. As a consequence of the mutation, DNMT1 protein is misfolded and degraded early after translation, it does not stay on the heterochromatin during G2 and mitosis, whereas its localization in S-phase is unchanged. In late S-phase DNMT1 loses the binding with UHRF1 and has impaired enzymatic activity [99, 100]. By analysing the global DNA methylation pattern, it has been shown that mutant DNMT1 induces hypomethylation of sat2, alpha-satellites, LINE and Alu repeats [99, 101]. Surprisingly, some gene promoters were found to be hypermethylated [99, 101]. This panorama is similar to the one present in cancer cells exhibiting global DNA demethylation and specific hypermethylation of some CpG islands at gene promoters (see “RE hypomethylation in cancer” paragraph); however, HSAN1E patients do not develop tumours [99].

Some differentially methylated genes are highly associated with neurological disorders [101]. However, it has not been established if their expression is altered in HSAN1E patients, or if the hypomethylation of REs allows unscheduled transcription like in ICF syndrome. It is, then, difficult to assess how and why the disease generates. Nevertheless, the importance of DNMT1 function in neuronal differentiation has been demonstrated by the generation of *DNMT1*-null mouse embryonic stem cells (ESCs) that cannot develop neuronal progenitors. Instead, these cells are prone to apoptosis, which might explain the observed degeneration of the nervous system [100]. Moreover, as we discuss in the “RE hypomethylation in neurodevelopmental, neurodegenerative, and psychiatric diseases” section, methylation levels at REs are important for brain development. However, considering that the symptoms of the disease appear from the second decade and worsen with age, and that DNMT1 loss of function results in a progressive and passive (from the missed DNA methylation at each cell division) DNA demethylation, it is tempting to think that the progressive global DNA methylation loss at REs is somehow the main inducer of the degeneration.

### Systemic lupus erythematosus

Systemic lupus erythematosus (SLE) (OMIM 152,700) is a complex autoimmune disease characterized by the uncontrolled production of autoantibodies, which causes inflammation and consequent potentially fatal damage in multiple tissues and organs [102]. Although it is known that the disease runs in families, the inheritance pattern is still unclear. Linkage studies identified the human leucocyte antigen (HLA) region as the strongest determinant of SLE, however, due to the variability of the disease, it has been proposed that the pathogenesis is determined by several factors including epigenetics (reviewed in [103]). In fact, already in 1990 it was discovered that T cells of SLE patients have hypomethylated DNA [104]. Interestingly, mice injected with murine CD4^+^ T cells, that were previously treated with 5 azacytidine—and thus demethylated—developed a lupus-like disease with production of anti-DNA antibodies, typical of SLE [105]. However, the fine mechanism by which hypomethylated T cells induce an immune response is still under investigation. Many promoter genes were found hypomethylated [103, 106], and, recently, also REs. In particular, long terminal repeats (LTRs), where the Human Endogenous Retrovirus-E (HERV-E) is embedded, have been found hypomethylated in CD4^+^ T cells of SLE patients. This also correlated with HERV-E transcriptional activation [107, 108]. Over the years, HERV expression has been proposed as the best candidate gene triggering the autoimmune response. This would suggest that the transcriptional activation of the LTR due to its hypomethylation could strongly influence the manifestation of SLE.

### Aicardi–Goutières syndrome

Aicardi–Goutières syndrome (AGS) (OMIM 225750) is a rare and highly variable genetic encephalopathy (120 cases known) characterized by inflammation and the consequent neurological dysfunctions, profound psychomotor retardation, basal-ganglial calcification, and cerebrospinal fluid lymphocytosis [109]. It is caused by mutations in one of the following genes: *TREX (three prime repair exonuclease 1), RNASEH2A, B, C (RNase H2 subunit A, B, C), SAMHD1 (SAM and HD domain-containing deoxynucleoside triphosphate triphosphohydrolase 1), ADAR1 (adenosine deaminases RNA specific)* and *IFIH1* (*interferon-induced helicase C domain-containing protein 1*), all involved in DNA and RNA metabolism. It is largely thought that the dysfunctional nucleases generate incompletely metabolized nucleic acid segments which trigger the activation of the immune response typical of AGS patients. However, although the research in the field has made considerable progress in the study of the molecular mechanisms that lead to the production of pro-inflammatory cytokines [110, 111], some pieces of the puzzle are still missing, such as the identity and origin of the immunogenic DNA sequences. Surprisingly, it has been recently discovered that, similarly to SLE, fibroblasts from AGS patients showed massive genome-wide DNA hypomethylation. This hypomethylation affected all genomic compartments including genic and intergenic regions and REs as well. In particular, LINEs, LTRs, SINEs and satellite repeats resulted to be significantly hypomethylated [112]. Moreover, genome-wide profiling of DNA:RNA hybrids showed that there is an accumulation of these hybrids exactly where the DNA has reduced methylation. It has also been demonstrated that the knockout of RNase H2A directly induces DNA methylation loss [112], suggesting that the epigenetic alteration can be at the base of the pathogenesis of AGS.

## RE hypomethylation in neurodevelopmental, neurodegenerative, and neuropsychiatric disorders

Recent advances in sequencing and genome analysis allowed the identification of multiple genotypes among the neurons in healthy adult brains [113]. This variability, or better, somatic mosaicism mainly arises from retrotransposon insertions which are thought to be central for the modulation of a transcriptional network involved in the neurodifferentiation and neuronal survival, and to contribute to the functional cellular diversity, memory formation and synaptic plasticity [114, 115]. However, the insertional preferences in some specific brain regions may influence the changes associated with healthy or diseased state. Thus, uncontrolled retrotransposition can cause deleterious genomic events jeopardizing the nervous system [116, 117]. To this regard, increasing observations have been made by associating brain diseases with abnormal expression of the TEs, mainly LINE-1 s although the causes of this alteration are not always clear. Undoubtedly, global hypermethylation represents a cautionary measure to limit the potentially harmful effects of TE’s jumping activity into nearby genes involved in vitally important neurological pathways [118, 119]. Several observations established that the activity and DNA methylation levels of LINE-1, the most studied type of REs, are subjected to dynamic temporal variations from the developmental period until ageing [120, 121]. REs indeed are thought to be sensors of environmental stress in the brain and may belong to a mechanism of programmed response to survive or counteract shocks or difficult conditions [122]. Although stressful stimuli are physiologically important to promote the neuroplasticity and reactivity of the hypothalamic–pituitary–adrenal (HPA) axis, the exposure to severe and prolonged stressors during the developmental age may induce maladaptive responses with long-term consequences in individual's physical and mental health [123]. The effect of environmental early life stress (ELS) may drastically change the DNA methylation pattern not only of genes involved in cognitive and behavioural functions, but also of TEs whose modulated activity might be correlated with major or minor susceptibility to negative events of every individual [122, 124]. Therefore, childhood adversities are recognized as significant risk factors for psychopathologies of the adult [125]. Accordingly, as reported below, the hypomethylation status of LINE-1 was prevalently observed in schizophrenia patients with a previous history of emotional trauma [126]. Moreover, in stressed children with high levels of cortisol, a well-known biomarker of chronic stress, a global decrease in DNA methylation was detected especially at SINEs and at binding sites of a zinc-finger transcription factor, ZNF263, having a function of repressor of retrotransposon activity [127].

Since REs are potential regulatory elements for brain development and functionality, their altered epigenetic regulation may represent a mutational signature for the risk assessment of neurological and psychological disorders. In this perspective, we addressed the variation in DNA methylation pattern of REs more commonly found in some neurodevelopmental, degenerative, and psychiatric disorders. We will not consider human diseases which showed RE aberrant expression but have not been studied from the DNAm point of view. In this case, we can only hypothesize that the overexpression results from RE hypomethylation, thus we preferred to exclude these diseases from this review, however we can suggest the interesting example of the Rett syndrome reviewed in [128].

### Ataxia telangiectasia

The decreased methylation of repetitive DNAs has been widely reported in ataxia telangiectasia (AT), a rare, neurodegenerative autosomal recessive disorder that occurs early in childhood and is characterized by a severe combined immunodeficiency and progressive cerebellar ataxia. AT is caused by homozygous or compound heterozygous mutations in the *ATM* gene encoding a serine/threonine protein kinase that plays a role in the control of double-stranded DNA break (DSB) repair, in the Purkinje cells of the cerebellum and in the brain cells. The impact of hypomethylation of LINE-1 and DNA satellites on chromosome stability was analysed in lymphocytes of AT patients [129]. The results suggested that the chromatin decondensation induced by DNA methylation loss—affected chromosomes are indeed elongated—promotes the chromosome rearrangements typical of patient cells. Likewise, an increased insertional activity of LINE-1 was found in postmortem brain samples of AT patients and *ATM* knockout mice [116, 130]. The above findings together suggest that the deregulation of LINE-1 may influence some of the symptoms and/or the progression of the disease, but it has not been associated with the aetiology of AT. However, the effect of LINE-1 hypomethylation is thought to be combined with the mutations in the ATM-dependent DNA repair signalling pathway although how the LINE-1 demethylation correlates with the ATM gene mutation is still to be examined [131].

### Autism spectrum disorders

Autism spectrum disorder (ASD) refers to a broad group of complex neurological and pervasive developmental disorders with high phenotypic heterogeneity ranging from mild, moderate-to-severe impairment of cognitive, language, behavioural and emotional skills. The wide clinical variability is thought to result from complex gene–environment interactions causing the susceptibility to ASD [132]. The main types of epigenetic RE-mediated dysregulation were detected in ASD include HERV, LINE-1 and Alu elements, collectively reviewed by [133]. However, up to now experimental evidence showed no significant alterations in RE methylation in ASD patients [134]. The only exceptions are the two subgroups of patients with severe language impairment and mild phenotype whose lymphoblastoid cell lines exhibited hypomethylation of LINE-1 s and Alus, respectively [135, 136]. Data concerning Alu hypomethylation revealed a pattern more specific for the ASD subgroup in which the milder symptoms associated with Alu upregulation [136]. First, Alu elements appear to be strongly associated with underexpressed genes in ASD inferring that Alu insertion most likely affects gene downregulation. Second, the specific position of Alu elements found in intronic regions of ASD-inserted genes implies that they have a role in the regulatory features of transcription, RNA splicing, and translation. Third, differentially expressed genes with Alu insertions are involved in the biological pathways prevalently associated with intellectual disability, cognitive impairment, and sex hormone-dependent signalling. In conclusion, the methylation analysis of REs needs further investigations to enable the development of an ‘epigenetic taxonomy’ of ASD subtypes improving the diagnosis and selective therapeutic strategies.

### Alzheimer’s disease

Alzheimer’s disease (AD) is the most common form of dementia characterized by the presence of abnormal beta-amyloid plaques and neurofibrillary tangles of Tau protein deposition which predominantly affects the entorhinal cortex and hippocampus. This causes progressive and irreversible memory loss and deterioration of cognitive functions impairing the daily function at advanced age (65–95 years of age) [137]. The major risk factor for AD is age since no underlying cause has been found yet. However, in the last decade overexpression of TEs—mainly Human Endogenous Retrovirus K (HERV-K)—has been observed in neural samples of Alzheimer’s patients and Drosophila models relevant to AD [138–140]. Notably, it has been demonstrated that Tau is sufficient to induce some TEs in Drosophila neurons, though this effect seems to be dependent on Tau functionality (wild type or mutant) and age [138, 140]. Surprisingly, it was also reported that HERV-K RNA, which is expressed in AD patients, can bind and activate Toll-like receptor 8 resulting in neurodegeneration and microglia accumulation. This evidence suggests that Tau-mediated neurodegeneration occurs through TE activation. Some studies discovered that the abnormal TEs activation in AD brain samples is triggered by a global decondensation of constitutive heterochromatin. The mutated Tau protein drives this chromatin destabilization and the loss of maintenance of neuronal integrity [139, 141]. However, not many analyses have been done to check if the relaxation of chromatin is associated with global DNA methylation loss. To our knowledge, no direct evaluation of HERV-K methylation has been made, thus we can hypothesize that the observed HERV-K over-expression is due to DNA hypomethylation. Instead, a decrease in the methylation of Alu and Satellite alpha repeats has been clearly assessed [142]. On the other hand, controversial data is available on DNA methylation of LINE-1. LINE-1 hypermethylation was found in peripheral blood leukocytes of AD patients, especially those with better cognitive performance, compared to healthy controls [142]. Nevertheless, other studies found no differences in LINE-1 methylation levels between AD patients and controls [143]. Recently, an interesting study identified differentially methylated LINE-1 and Alu elements of specific loci in elder people with type 2 diabetes who are most likely to develop pre-symptomatic dementia (PSD). These loci were enriched in genes involved in AD and cognitive decline. Surprisingly, dietary supplementation with folate and vitamin B12, which contribute to S‐adenosyl‐methionine (donor of methyl groups) production, changed methylation pattern suggesting that it could potentially lower the dementia risk [144].

### Bipolar disorder

HERV elements are the most characterized REs in bipolar disorder (BP), a mental health condition that causes extreme mood swings. Several studies reported the overexpression of HERV-K and HERV-W env sequences in brain and blood samples of BP patients, respectively [145, 146]. However, if this overexpression is due to DNA hypomethylation has not been assessed yet. Some preliminary results are, instead, available about the hypomethylation status of LINE-1 and of AluY subfamilies that were examined in patients with BP and schizophrenia (as also reported in the next paragraph). Hypomethylation of two out of three analysed CpG sites of LINE-1 repeats (S2 and S3) was observed in the peripheral blood of BP patients [147]. Moreover, AluY subfamily A3 was found to be hypomethylated at A3 CpG site—though hypermethylation of subfamilies A1 and A2 was also reported—in the peripheral blood DNA of the patients [148]. How RE hypomethylation and presumably activation could be related to the appearance of the disease is still unexplored.

### Schizophrenia

Schizophrenia (SZ) is a severe neuropsychiatric disorder comprising cognitive, behavioural, and emotional disturbances, whose most common psychotic symptoms are hallucinations, disorganized thinking, and abnormal motor behaviour. Despite extensive research, the cause of SZ is still elusive. Heritable factors (gene-expression profiles, single-nucleotide variants) have been found that confer a high risk of SZ. However, most probably environmental factors concur with the genetic background for the development of the disease [149]. In this view, epigenetic modifications, the litmus paper of environmental event, could play a role in this complex disease. A trend towards hypomethylation is suggested by the observation that in the prefrontal cortex neurons of patients there are many LINE-1 insertions in genes involved in synaptic function and neural circuits evolution [150, 151]. Findings of the LINE-1 methylation status analysed in peripheral blood leukocytes of individuals with SZ are, however, mixed. The hypomethylation of LINE-1 subfamilies S1 and S3 was observed in SZ patients [147]. The level of LINE-1 methylation was found even lower in those SZ patients with a history of childhood trauma compared to other SZ patients [126]. On the other hand, other studies revealed a higher LINE-1 methylation in patients with first-episode psychosis, or partial methylation in paranoid SZ, and methamphetamine-induced paranoia [152, 153].

As discussed previously for BP disorder, the study of Li et al. [148] on distinct methylation patterns of AluY elements, has also revealed the hypermethylation status of the A2 CpG site in SZ as opposed to BP patients, whereas the hypomethylation of A3 CpG site was more pronounced in SZ than BP patients.

### Major depressive disorder

Major depressive disorder (MDD) is a mental disorder characterized by low self-esteem, guilt, pervasive sadness, and loss of interest. In some cases, it is also associated with anxiety anger and irritability [154]. Some genetic variants are known to put some individuals at higher risk to experience the disease [155], although it also documented that MDD depends on the combination of genotype, brain chemistry and the interaction with other cumulative effects [156]: familial cases of depression, inheritance of temperament and maladaptive character traits, manifestation of chronic health problems, alcohol and substance abuse, social (i.e. low income, poor quality of life, social exclusion) and environmental determinants such as chemical pollutants, natural disasters and trauma, and both physical and emotional. However, it still needs to be clarified how the environmental stress can interact with or impact the genetic factors. Some evidence suggested a role for the hypomethylation of REs. Indeed, the increased copy number of LINE-1 was coupled with hypomethylation of these REs in the peripheral blood of MDD patients [157]. In addition, hypomethylation occurs in the AluJb element inside the promoter of the serotonin transporter *SLC6A4* gene, which is the main target of the antidepressant medications used in MDD therapy [158]. Notably, the AluJb methylation assessed in blood samples was even lower in stressed compared to less stressed individuals, positively associated with amygdala reactivity to emotional faces but negatively with depression [158]. The contribution of all these factors can lead to detrimental effects of genome instability underlying neuropsychiatric dynamics in MDD patients compared to healthy subjects.

### Post-traumatic stress disorder

People who have experienced or witnessed a painful and traumatic event, such as car accident, natural catastrophes, terroristic attack, war, rape, or child abuse may develop an anxiety disorder termed as post-traumatic stress disorder (PTSD) [159]. The epigenetic contribution in the PTSD development by exposure to ELS has been widely documented [160]. The existence of an epigenetic inheritance program that implies the risk of PTSD transmission in the offspring (intergenerational effects) was observed in the progeny of holocaust survivors and more recently, in the survivors of the World Trade Centre attack [161, 162]. However, few data are available that estimate the role of REs in PTSD. There is only one study analysing DNA methylation of REs in the serum of military personnel diagnosed with (cases) and without (controls) PTSD after deployment. This research showed that soldiers more resilient (controls) were associated with LINE-1 and Alu hypomethylation compared to cases [163]. Thus, hypomethylation of REs seems to be involved in the response to psychological stressors. However, the study of RE methylation in PTSD is quite recent, therefore, it is yet difficult to understand its role in the pathogenesis of the disorder, if one actually exists.

## RE hypomethylation in cancer

In 1983 Feinberg and Vogelstein, for the first time, found, by Southern blotting of DNA digested with methylation-sensitive restriction enzymes, that cancer cell genome is extensively hypomethylated with respect to cells from normal tissues [164]. The same observation was made by the Ehrlich group by using liquid chromatography [165]. Every tumour analysed so far exhibits loss of DNAm as a common feature [166]. However, hypermethylation of some CpG islands at gene promoters coexists with the “global” hypomethylation in cancer cells [167], a condition shared with rheumatoid arthritis, an autoimmune and inflammatory disease with a high incidence of cancer [168]. This ostensible paradox comes out from the fact that usually the methylation, rather than CpG islands, takes place at the repetitive sequences which, as discussed above, constitute the vast majority of the human genome [19, 21]. In cancer, where DNAm is deregulated (both hypo- and hyper-methylation that probably arise independently [167]), the DNA hypomethylation impacts a larger part of the genome than DNA hypermethylation does. Consequently, the net amount of 5-methyl-cytosines in the genome is decreased with respect to normal tissues. While the role of hypermethylation and hypomethylation at gene promoters’ CpG islands in cancer is intuitive through its effect in silencing tumour suppressor genes and in activating oncogenes, respectively, the role of RE hypomethylation is still elusive, though it is a regular event in tumour cells. Virtually all kinds of repeated sequences undergo loss of methylation in cancer (summarized in [169]). We will next review the most recurrent hypomethylated REs in cancer.

### Interspersed repeat hypomethylation in cancer

LINE-1 hypomethylation closely correlates with global hypomethylation [169] and is highly recurrent in the majority of cancer types (hepatocellular carcinoma, extra-hepatic cholangiocarcinoma, bladder cancer, chronic lymphocytic leukaemia, gastrointestinal stromal tumour, colon cancer, lung carcinoma, ovarian carcinoma) [170–179]. Intriguingly, it has often been observed that LINE-1 hypomethylation increases together with the histological grade of the tumour and a poor prognosis especially in gastrointestinal cancers [174, 176, 179, 180]. Moreover, the hypomethylation seems to be specific of the tumoural tissue and is not found in the premalignant lesions or in the peripheral blood which is the case for other hypomethylated REs such as satellite 2 [171, 175]. Given this peculiarity, the degree of methylation of LINE-1 interspersed repeats has been thought of for its potential use in cancer diagnosis and prognosis.

Other interspersed repeats frequently demethylated in tumours are the Alu repeats (hepatocellular carcinoma, bladder cancer, lymphocytic leukaemia, gastrointestinal stromal tumour, thyroid tumour) [170, 172–174, 181]. An interesting clinical study on thyroid tumours recently showed that, by using the quantification of unmethylated Alu (QUAlu) technique, distant metastatic differentiated thyroid cancer (DTCs), poorly differentiated thyroid cancer (PDTCs) and anaplastic thyroid cancer (ATCs) are characterized by an increased hypomethylation of the Alu repeats [181]. Interestingly, these three types of thyroid tumours have a poor prognosis compared to other kind of tumours (e.g., paediatric DTCs) which show no Alu hypomethylation. This is also coupled with an increase in cell de-differentiation, strongly suggesting that Alu hypomethylation could be associated with the advanced stages of the disease. However, this study failed in finding a worsening of hypomethylation in spread distant metastases, meaning that this alteration is stable once established [181].

It is noteworthy that the relationship between age and cancer about interspersed repeat hypomethylation is controversial. Indeed, it has been observed that the global hypomethylation correlates with age (regardless cancer progression) in gastric cancers, suggesting that hypomethylation naturally occurs (see “RE hypomethylation and ageing” paragraph) and predisposes to genome instability and thus to cancer transformation [182]. In contrast, in thyroid tumours no correlation between age and hypomethylation of Alu repeats has been found, hinting that hypomethylation is more dependent on cancer risk rather than on ageing [181].

### Tandem repeat hypomethylation in cancer

Tandem repeats, particularly centromeric alpha-satellites, juxtacentromeric sat2 and NBL2 repeats which are close to the centromere of most acrocentric chromosomes, are significantly hypomethylated in a wide variety of tumours (neuroblastoma, hepatocellular carcinoma, breast, bladder, ovarian and gastrointestinal cancers, Wilms tumours) [172–174, 183–191]. Widschwendter’s and Ehrlich’s groups also showed that the hypomethylation of sat2 and alpha-satellites (especially of chromosomes 1 and 16) are closely correlated with an advanced stage of ovarian epithelial tumour [189, 190]. This suggested that such hypomethylation patterns can be of prognostic significance for a specific kind of cancer. Interestingly, hypomethylation of satellite 2 has been associated with rearrangements involving the pericentromere, such as the gain of chromosome 1q in hepatocellular carcinoma [186]. Similarly, in Wilms tumours the hypomethylated alpha-satellites of chromosome 16 and 1 strongly correlated with the loss of chromosome 16q and the gain of chromosome 1q, respectively [191]. This evidence clearly highlights how the maintenance of the methylation degree of REs protects from chromosome fragility. In fact, methylation loss has been directly correlated with satellite decondensation, specifically satellite 3, with transcription activation in tumour and in aged cells as well (see “RE hypomethylation and ageing” paragraph), and with centromere alterations [49, 192]. It is also noteworthy that lncRNAs have been found to arise from hypomethylated tandem repeats in cancer. This is the case of the tumour-associated NBL2 transcripts (TNBLs) observed in colon cancer [188]. In colon cancer, indeed, NBL2 pericentromeric repeats are demethylated in chromosomes 21, 7 and 9, allowing transcription of the subtended sequences, precisely the TNBLs, which form aggregates at the perinucleolar region and can bind proteins involved in nuclear functions and RNA metabolism (namely CELF1 and NPM1) [187, 188]. Thus, TNBLs have the potential to impair other RNA functions and to impact nuclear architecture. If and how this is linked to tumour initiation/progression is still under investigation. In addition, it has been demonstrated that *HELLS* downregulation is able to cause NBL2 hypomethylation [187]. Curiously, *HELLS* is also mutated in ICF syndrome [61] (see the “Immunodeficiency with centromeric instability and facial anomalies syndrome” section).

### Hypomethylation of REs and its potential contribution to carcinogenesis

**Fig. 2:**
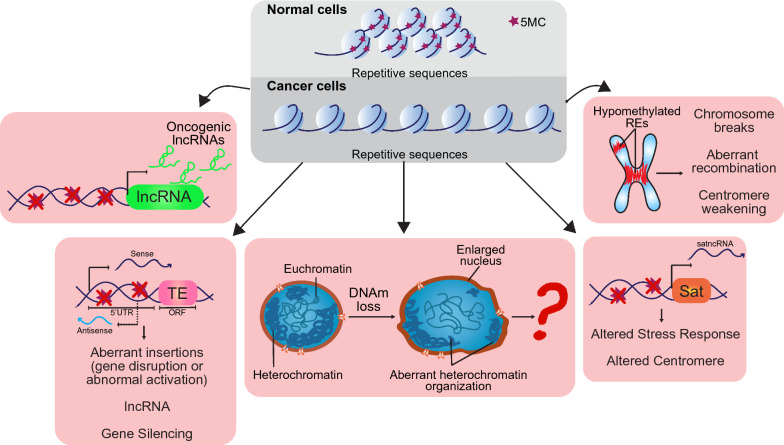
Potential contributions of hypomethylated REs to carcinogenesis. As opposed to normal cells, cancer cells are characterized by cytosine methylation loss at repetitive DNA. This alteration can affect cell behaviour and contribute to cancer initiation/progression in several ways. The hypomethylated REs can be regulators of oncogenic lncRNAs and, thus, induce their abnormal transcription. TEs or satellite DNA, once hypomethylated, can be also transcribed potentially affecting several processes and leading to genomic and chromosome stability. Furthermore, hypomethylation of REs could affect chromosome structure making it more fragile and prone to breaks, recombination and even to the weakening of centromere function. By changing the compaction degree of the chromatin, hypomethylation of REs also affects nucleus size and organization which, we believe, could dangerously compromise cells, though this research field has not been well explored

While it is a staple that cancer cells undergo DNA methylation loss at repetitive DNA sequences, it is not clear if this has a univocal role in tumorigenesis or not. However, several hypotheses have been formulated and explored. It is indeed widely accepted that the DNA decondensation consequential to RE hypomethylation has an impact on multiple cell functions, namely on gene expression, DNA repair, recombination, and chromosome segregation. Alterations of these events can be deleterious and drive or worsen cancer progression (Fig. 2).

Experimental results showed that impairment of DNMTs and DNA demethylation caused genetic instability and aneuploidy [49, 193–195]. Remarkably, we showed that in primary fibroblasts DNMT1 downregulation triggers a cell response that blocks cell cycle thus avoiding DNAh [193]. This response is dependent on p14ARF, which has been shown to protect cells from aneuploidy [196–198]. Therefore, DNA methylation loss is seen by cells as a potential danger for chromosome dynamics. In fact, specific loss of methylation at peri-/centromeric region is accompanied by aneuploidy and chromosome rearrangements [49, 186, 191], suggesting that the epigenetic status of this region is essential to maintain centromere integrity and faithful function. It is likely that the hypomethylated DNA results in more fragility increasing the possibilities of recombination [33]. Nevertheless, the fine mechanism by which DNAm sways centromere function is still an open question.

Hypomethylation of interspersed repeats has also been correlated with genome instability [169, 199], however it has been difficult to demonstrate a direct effect. Recently, it has been shown that methylation of Alu repeats is negatively correlated with DNA damage lesions in peripheral blood cells. Also, it was demonstrated that the hypermethylation of these repeats confer a higher resistance against different DNA damaging agents and by itself reduce the concentration of DNA lesions in comparison to hypomethylated cells [200]. This evidence indirectly suggests that hypomethylation of Alu repeats would put a strain on genome stability. How this is exerted is still unclear. One hypothesis is that DNA hypomethylation activates the TEs, which can contribute to chromosome rearrangements and also induce oncogene activation similar to what was observed in murine tumours with the hypomethylation-induced insertion of intracisternal A particles into the Notch locus [201]. In the last 10 years Tufarelli’s group has been studying the role of LINE-1s chimeric transcripts (LCTs) that originate from the hypomethylation-induced activation of antisense promoter in LINE-1s. This study took into account several tumours such as breast and colon cancers [202]. These lncRNAs have been found to modulate gene expression in their surrounding and over large distances. They can both silence tumour suppressor genes and activate oncogenes thus contributing to cancer [203].

Besides, it should not be forgotten that DNA methylation immediate result is chromatin compaction. Therefore, a global DNA hypomethylation would impact the whole chromatin 3D structure affecting nucleus size and spatial organization. In this regard, some years ago an interesting work showed how hypomethylation of REs—namely LINE-1, satellite 2 and alpha satellite—is strongly correlated with the nuclear size and consequently with chromosome aneuploidy in ovarian cancers [204]. The architecture of chromosomes in the nucleus is still quite nebulous, but we do know that some heterochromatic regions occupy specific places in the nucleus by associating with the nuclear lamina (LADs, nuclear lamina-associated domains) [205]. The LADs are defined and confined due to DNA methylation’s special pattern and it should not come as a surprise that they coincide with REs [206]. As a consequence, LADs are completely mixed up in cancer cells [207, 208]. Thus, the DNAm changes in cancer induce a reorganization of both heterochromatin distribution and nuclear architecture. We can speculate that this can somehow control not only gene expression, but also nuclear behaviour, promoting genome instability.

## RE hypomethylation and ageing

We reviewed many studies highlighting a role for RE hypomethylation in hereditary and neurological diseases and in cancer as well. However, the number of human diseases where this alteration has been found is increasing, such as atherosclerosis and cardiovascular diseases, metabolic syndromes, rheumatoid arthritis, and Fanconi anaemia [129, 209–211]. Due to space constraints, we will not address all of them. However, we want to discuss here the subject of ageing, since the frequency of some of these diseases is correlated with age.

In the same year when Feinberg and Vogelstein published their work on cancer, other groups showed how DNAm is progressively lost during ageing in mice and humans [212–214]. This effect was observed in both cells extracted from the organisms at different ages and in cells cultured in vitro up to the appearance of the senescence. To this regard, as discussed above, it has been observed that the methylation level of satellite 3 is lost in human fibroblasts (MRC5 and VH-10) at a late passage [192]. Consistent with this, it was demonstrated that chromatin of senescent fibroblasts is mainly open, and heterochromatin is intensively lost compared to early passages. Moreover, Alu, LINE-1 and satellite repeats become more open and active in senescent cells [215]. This observation has been confirmed with up-to-date techniques which showed, in particular, the hypomethylation of Alu repeats in the blood cells of healthy volunteers at increasing ages [216, 217]. Interestingly, the study by Gentilini et al. demonstrated that age-related global hypomethylation was less prominent in the offspring of centenarians, and that there is no significant change in Alu methylation in centenarians’ offspring with respect to controls of young age. This suggests that a better preservation of DNA methylation could contribute to the process of human longevity [218]. The pattern of LINE-1 methylation has been controversial for years, suggesting a different regulation. Two years ago, an interesting study was published showing that in fact hypomethylation of both Alu and LINE-1 REs is an epigenetic marker of ageing, though not in blood cell DNA, but in the circulating cell-free DNA [219]. By using idiolocal normalization of real-time methylation-specific PCR (IDLN-MSP), the authors could analyse the methylation status of Alus and LINE-1s in the cell-free DNA of a cohort of healthy volunteers from 20 to 60 years of age. The correlation between age and DNA methylation of LINE-1 and Alu REs of cell-free DNA is impressive. Unfortunately, the molecular origin of the cell-free DNA in human body fluids remains poorly understood and thus we can only conjecture on the implication of its hypomethylation. The most accredited hypothesis is that in healthy individuals this DNA derives from cell death and clearance in multiple regions of the body [220]. Thus, it is likely that the observed hypomethylation at REs of this DNA is proof of the genomic instability driving cell death which increases with age.

## Conclusions

Repetitive DNA sequences are a fascinating research topic that has been quite snubbed and considered for a long period as the “junk”, though a substantial (two-thirds of the genome) part of the genome. Instead, over time, it is becoming evident that these sequences cannot be just useless, rather they have a central role in the regulation of the human genome with involvement in virtually all cell processes. Such an important role, thus, must be carefully directed to avoid errors and potential pathologic outcomes. Here, epigenetics comes into play, and in particular DNA methylation. Indeed, due to their intrinsic repetitive nature, these DNA elements are difficult to replicate and prone to recombine, in addition many of them have transposable activity and can be inserted genome-wide. All these can jeopardize genome stability. Therefore, REs are mostly repressed by DNAm. It is not surprising, then, that DNA hypomethylation of basically every kind of RE has been associated with a multitude of human diseases, from brain disorders to cancer (Table 1). Here, we reviewed the literature that shows evidence of RE hypomethylation in several human diseases. It appears that RE methylation is a key element that allows proper cell behaviour, correct development and ageing. Considering that the majority of 5MCs of the whole genome are inside the REs, the impairment of DNA methylation—at least as passive methylation loss—would affect primarily these important sequences altering their role in the genome. Thus, the preservation of the correct level of global DNA methylation could be essential for healthy cells and individuals. The analysis of DNA methylation profiles of REs tends to be appealing as it allows for an arsenal of biomarkers to disentangle the high variability of neurological and cancer phenotypes improving the clinical diagnosis and choice of treatment options of patients’ subtypes, which are still a major challenge for unmedicated cases. Although more research is needed to better determine types of actions elicited by DNAh in different REs for a protective or toxic effect in the brain, current achievements are promising to predict how these different activities can be counterbalanced. In this regard, recent studies have highlighted the emerging role in immuno-oncology of the reactivation of TEs by the therapeutic administration of demethylating agents as decitabine [221–223]. Demethylating drugs, in fact, have been used for almost two decades in cancer treatment [224] In addition to this role, demethylating agents, through reactivation of TEs, have been more recently described as inducers of innate immunity [221, 225] and promoters of the immunosurveillance by enhancing antiviral signalling and the production of neoantigens in tumour cells [222, 223].

**Table 1:** Hypomethylated repetitive elements in human diseases

	Disease	Type of RE	Cell type	References
Hereditary diseases	AGS	LINEs, LTRs, SINEs and DNA satellites	Fibroblasts	Lim et al. [112]
	ICF	Sat1 and 2	Fibroblasts, lymphoblasts	Hassan et al. [57]
			Leukocytes	Jeanpierre et al. [54]
			Lymphocytes	Ji et al. [56], Miniou et al. [55], Heyn et al. [58], Simo-Riudalbas et al. [72]
		Sat-α	Fibroblasts, leukocytes	Miniou et al. [63]
			Blood cells	Velasco et al. [62]
		Sub-telomeric repeats	Lymphoblastoid cell lines	Sagie et al. [67]
		D4Z4 and NBL2		Kondo et al. [71]
	FSHD	D4Z4 and NBL2	Several tissues including blood	van Overveld et al. [84], de Greef et al. [81], Lemmers et al. [85], Gaillard et al. [87]
	HSAN1E	Sat2, sat-α, LINEs and Alus	Blood cells	Klein et al. [99], Sun et al. [101]
	SLE	HERV-E	Blood cells	Wu et al. [107], Wang et al. [108]
Cancer	Breast cancer	Sat2, sat-α, LINEs and Alus	Tumoural cells	Narayan et al. [183]
	Bladder cancer	Sat2, LINE-1 and Alus, sat-α, NBL2	Tumoural cells	Si et al. [172]
	CLL	Alus, LINEs, LTRs, sat	Tumoural cells	Subhash et al. [173]
	Colon cancer	NBL2	Tumoural cells	Samuelsson et al. [187], Dumbović et al. [188]
		LINE-1	Blood cells	Samuelsson et al. [187], Suter et al. [175]
	Extra-hepatic cholangiocarcinoma	LINE-1, sat2	Blood cells	Kim et al. [171]
	Gastrointestinal stromal tumour	AluYb8, sat-α, NBL2	Tumoural cells	Igarashi et al. [174]
		LINE-1		Igarashi et al. [174], Shigaki et al. [179]
	Hepatocellular carcinoma	Alus and LINEs	Tumoural cells	Zheng et al. [170]
		Sat2		Wong et al. [186]
		NBL2		Nagai et al. [185]
	Neuroblastoma	NBL2	Tumoural cells	Thoraval et al. [184]
	Lung carcinoma	Alu	Tumoural cells	Ikeda et al. [176]
	Ovarian carcinoma	LINE-1	Tumoural cells	Notaro et al. [177], Zhang et al. [178]
		sat2 and sat-α	Tumoural cells	Qu et al. [191], Widschwendter et al. [190]
	Thyroid cancer	Alus	Tumoural cells	Hesselink et al. [181]
	Wilms tumours	Sat2 and sat-α	Tumoural cells	Qu et al. [189]
Ageing	Ageing	Sat3	Fibroblasts	Enukashvily et al. [192]
		Alus	Blood cells	Bollati et al. [216]
				Jintaridth and Mutirangura [217]
		Alus and LINE-1	DNA-cell free	Erichsen et al. [219]
Brain diseases	Neurodevelopmental disorders			
	ASD	LINE-1 s	Lymphocytes	Tangsuwansri et al. [135]
		Alus		Saeliw et al. [136]
	AT	LINE-1, sat2, sat-α	Lymphocytes	Almeida et al. [129]
	Neurodegenerative disorders			
	AD	Alus, sat-α	Leukocytes	Bollati et al. [142]
	Neuropsychiatric disorders			
	BP	LINE-1 S2 and S3	Blood cells	Li et al. [147]
		AluYA3		Li et al. [148]
	ELS-related disorders	LINE-1	Leukocytes	Misiak et al. [126]
		SINEs		Nätt et al. [127]
	MDD	LINE-1	Blood cells	Liu et al. [157]
		AluJb		Schneider et al. [158]
	PTSD	Alus and LINE-1	Serum	Rusiecki et al. [163]
	SZ	LINE-1	Blood cells	Misiak et al. [126]
		LINE-1 S1 and S3		Li et al. [147]
		AluYA3		Li et al. [148]

*AGS* Aicardi–Goutières syndrome, *AD* Alzheimer's disease, *ASD* autism spectrum disorders, *AT* ataxia teleangectasia, *BP* bipolar disorder, *CLL* chronic lymphocytic leukaemia, *ELS* early life stress, *ICF* immunodeficiency with centromeric instability and facial anomalies syndrome, *FSHD* facioscapulohumeral muscular dystrophy, *MDD* major depressive disorder, *PTSD* post-traumatic stress disorder, *SZ* schizophrenia

New findings about the DNA hypomethylation mechanism, molecular and cellular consequences, DNAm preservation and not lastly about tools/strategies to study and modulate this epigenetic modification would be undoubtedly helpful to understand more and provide therapies for many human diseases.

## Data Availability

Not applicable.

## Abbreviations

AD: Alzheimer’s disease
*ADAR1*: Adenosine deaminases RNA specific
AGS: Aicardi–Goutières syndrome
ASD: Autism spectrum disorder
AT: Ataxia telangiectasia
ATCs: Anaplastic thyroid cancer
*ATM*: ATM serine/threonine kinase
Aza: 5 Azacytidine
BP: Bipolar disorder
*CDCA7*: Cell division cycle-associated protein 7
DNAh: DNA hypomethylation
DNAm: DNA methylation
*DNMT3B*: DNA methyltransferase 3 beta
DNMTs: DNA methyltransferases
DTCs: Differentiated thyroid cancer
ELS: Early life stress
ESCs: Embryonic stem cells
FSHD: Facio-scapulo-humeral muscular dystrophy
FSHD2: Phenotypic FSHD patients
H3K9 me3: H3K9 trimethylation
HELLS: Helicase, lymphoid specific
HERV-E, K, W: Human Endogenous Retrovirus-E, K, W
HLA: Human leucocyte antigen
HP1: Heterochromatic protein 1
HSAN1E: Hereditary sensory and autonomic neuropathy type 1E
ICF1-4: ICF subtype 1,2,3,4
IDLN-MSP: Idiolocal normalization of real-time methylation-specific PCR
IFC: Immunodeficiency with centromeric instability and facial anomalies syndrome
LADs: Lamina-associated domains
lncRNAs: Long noncoding RNAs
LTRs: Long terminal repeats
MDD: Major depressive disorder
PDTCs: Poorly differentiated thyroid cancer
PTSD: Post-traumatic stress disorder
QUAlu: Quantification of unmethylated Alu
REs: Repetitive elements
*SAMHD1*: *SAM And HD Domain Containing Deoxynucleoside Triphosphate Triphosphohydrolase 1*
Sat1-3: Satellite 1,2,3
SLE: Systemic lupus erythematosus
SMCHD1: Structural maintenance of chromosomes flexible hinge domain-containing 1
SSRs: Simple sequence repeats
SZ: Schizophrenia
TEs: Transposable elements
TNBLs: Tumour-associated NBL2 transcripts
*TREX*: Three prime repair exonuclease 1
TS: Targeting sequence
*UHRF1*: Ubiquitin like with PHD and ring finger domains 1
VNTRs: Variable number of tandem repeats
*ZBTB24*: Zinc finger and BTB domain containing 24
